# Detection of depression risk among older adults using home-deployed socially assistive robots: a real-world study

**DOI:** 10.3389/fpsyt.2026.1740539

**Published:** 2026-06-10

**Authors:** Han Wool Jung, Jooho Lee, Jin Young Park, Woo Jung Kim, Jaesub Park

**Affiliations:** 1 Department of Psychiatry, Yongin Severance Hospital, Yonsei University College of Medicine, Yongin, Republic of Korea; 2 Department of Integrative Medicine, Yonsei University College of Medicine, Seoul, Republic of Korea; 3 Digital Medic Co., Ltd., Seoul, Republic of Korea; 4 Institute of Behavioral Science in Medicine, Yonsei University College of Medicine, Seoul, Republic of Korea; 5 Center for Digital Health, Yongin Severance Hospital, Yonsei University College of Medicine, Yongin, Republic of Korea

**Keywords:** digital phenotyping, ecological momentary assessment, passive sensing, precision psychiatry, real-world data

## Abstract

**Introduction:**

Monitoring depression among older adults using socially assistive robots provides scalable and continuous health surveillance while reducing the clinical burden on therapists and minimizing delays in treatment. This study aimed to predict depression risk and identify individuals in need of specialized depression care at local healthcare centers, using response and usage log data from the socially assistive robot *Hyodol*.

**Methods:**

A total of 215 community-dwelling older adults (170 in the 2024 cohort and 45 in the 2025 cohort) who used *Hyodol* were recruited. User responses to *Hyodol*’s daily health check-in questions and free conversations, as well as physical interaction and content usage logs, were processed as features. Depression status was determined via clinical surveys and expert-led video consultations. A random forest model to predict depression status, defined as (i) symptomatic of depression and (ii) depression requiring referral to local healthcare centers, was trained on the 2024 cohort and tested on the 2025 cohort.

**Results:**

The model predicted symptomatic participants and participants requiring referral with sensitivities of 0.939 and 0.900, respectively. The model also produced considerable false positives. Features most strongly associated with depression status included engagement with quiz content, frequency of free conversations, positive responses to daily check-ins, regular meal intake, and the frequency of physical interactions with the robot.

**Discussion:**

The preliminary findings suggest that *Hyodol*-based monitoring may serve as a viable screening tool for detecting depression risk in older adults. Future work should focus on refining the model to replicate current results while minimizing false alarms, incorporating more in-depth content analysis, and developing continuous emergency monitoring and alert systems to enhance clinical utility.

## 1 Introduction

Older adults require home-based mental healthcare in addition to standard outpatient treatment, as mobility limitations, physical health problems, and social isolation often hinder their access to clinic-based services (1). Home-based care not only addresses these challenges but also provides more personalized, context-sensitive, and patient-centered interventions that can improve mental health outcomes and treatment adherence among older adults (2, 3). Recently, the use of socially assistive robots (SARs) in home-based care has emerged as a promising approach for geriatric mental healthcare. SARs can address the issues of staffing costs and visitation time in traditional in-person home care while enabling continuous, around-the-clock monitoring for multiple individuals with minimal human caregiver involvement (4–6).

Many studies suggest that SARs are well accepted by older adults and are effective in alleviating mental health symptoms such as depression, loneliness, and mild cognitive impairment. *Hyodol*, a SAR designed with the persona of a grandchild, provides highly engaging, patient-centered daily care for older adults and has demonstrated both high user acceptance and clinical efficacy (7–10). Other SARs, such as *PARO*, have also shown promising results in improving the mental health of older adults in home-based and daycare settings (11, 12). However, most studies on robots for older adult care have predominantly focused on short-term clinical outcomes without developing or validating patient health monitoring functions, which are also recognized as a critical component of SARs (4, 13, 14). Health monitoring with SARs can further reduce the burden on human caregivers while promoting independent living and the quality of life of older adults (15, 16).

Integrating monitoring functions into SARs requires the comprehensive incorporation of technologies such as the Internet of Things (IoT) and artificial intelligence (AI) for effective sensing (5). Leveraging IoT capabilities allows SARs to continuously capture users’ activity and emotional patterns, which can be further utilized in the development of integrated systems for intensive care or virtual social communities (17). Robot-based or robot-linked passive sensing enables objective, continuous detection of patient status, facilitating early identification of mental health risks (15, 18, 19). Advances in medical AI have further enabled the accurate prediction of mental health symptoms by combining passive and active sensing data with behavioral indicators, such as speech, movement, or facial expressions, even in real time (digital phenotyping). This integration allows for automatic and immediate alerts in emergencies, including suicidal crises (18–20). Finally, this framework is evolving into the “Internet of Robotic Things,” which combines SARs with ambient sensors to support independent living among older adults, and the “Internet of Medical Things,” which integrates SARs, IoT sensors, and AI systems for real-time symptom detection and just-in-time interventions for mental health symptoms (16, 21).

The present study is a preliminary investigation aimed at developing an AI-based detection model to identify older adults at risk of depression through the SAR *Hyodol*, using both active and passive usage data collected from the robot. By combining responses to daily health check-ins and free-form interactions with real-world usage logs, this study developed a model capable of classifying users at risk of depression and identifying those requiring specialized care at local healthcare centers, as determined by clinical surveys and remote clinical assessments. Once integrated into *Hyodol*, the robot will function as an automated screening tool, capable of proactively identifying users at risk of depression, thereby promoting the well-being of older adults and potentially reducing medical costs within the community.

## 2 Methods

### 2.1 Participants

The participants were community-dwelling older adults aged 65 or older who had been continuously using *Hyodol* at home and consented to participate in the study aimed at developing a *Hyodol*-based remote healthcare service model. All participants resided in rural areas, had no spouse (unmarried or bereaved) or lived alone, and were affiliated with local welfare centers providing care services for older adults. Informed consent included participation in an initial clinical survey, potential involvement in remote video interview sessions based on survey results, and permission to collect and use personal and sensitive data for research purposes, including demographic and contact information, device identifiers, location and daily routine data, voice data, sleep, pain, and mood status, medication- and activity-related records, and *Hyodol* response and usage logs. The study was approved by the Institutional Review Board of Yongin Severance Hospital (IRB study number: 9-2024-0031; date of approval: April 19, 2024), and the clinical effects of the *Hyodol*-based intervention in this dataset were previously reported in Jung et al. (2026) (22).

### 2.2 Data acquisition from *Hyodol*

*Hyodol* continuously collected and recorded user responses and activity logs during daily use. For daily health check-ins, the robot prompted users to report on their current mood, pain, sleep quality, meal status, and medication status through voice interactions, recording their responses twice daily, once in the morning and once in the afternoon. User responses were transcribed into text using a speech-to-text algorithm and subsequently classified as positive, neutral, or negative through zero-shot sentiment analysis performed by a GPT-based custom large language model (LLM). The frequency of responses to health check-in questions (relative to skipped questions), along with daily meal and medication status, was also documented as part of the daily usage records.

Participants could also engage in free-form conversations with *Hyodol* via voice interactions. These conversational features were powered by a GPT-based custom LLM, incorporating real-time interruption capability to enable natural, human-like dialogue. In addition to the voice and text-converted responses themselves, the frequency of free interactions was recorded as part of the daily usage logs. Other usage data included the number of instances of patting, tapping, and holding hands with *Hyodol* (detected via embedded sensors), as well as frequency of content usage such as Bible or Buddhist scripture readings, storytelling, retro pop songs, English lessons, remembrance games, classical and religious music, exercise routines, quizzes, and hearing function checks. All participants used *Hyodol* for at least 4 weeks (minimum of 27 days). All data were collected with written informed consent from all participants and were used only for research and analysis. For analysis and data sharing, records were handled in de-identified form using coded identifiers, and directly identifiable information was not included in research datasets provided to authorized parties. Access to study records was restricted to authorized personnel and could be granted to regulatory reviewers as permitted by applicable regulations.

### 2.3 Clinical survey and video consultation procedures

Participants who consented to the study completed an initial clinical survey for screening. The survey included the Geriatric Depression Scale – Short Form (GDS-SF) (23), the Patient Health Questionnaire-9 (PHQ-9) (24), the WHO Disability Assessment Schedule 2.0 – 12-item (WHODAS) (25), the UCLA Loneliness Scale – Version 3 (26), and a custom health management checklist. Participants who met the criteria for depression or related problems on any screening scale were invited to a first video interview session, whereas those who did not were classified as non-depressed. Specifically, the criteria included: a GDS scoring ≥ 6, indicating depressive symptoms requiring further psychological assessment (27); a PHQ-9 score ≥ 5, indicating the presence of depressive symptoms (24, 28); a PHQ-9 item 9 > 0 (“not at all”), indicating suicidal thoughts (29); a UCLA Loneliness Scale ≥ 65, reflecting high level of loneliness (30, 31); a WHODAS ≥ 24, indicating poor functional ability (32); and a health management checklist score ≤ 10, indicating reduced health management capacity.

The first video interview session consisted of a structured clinical interview using the depression module of the Mini-International Neuropsychiatric Interview 7.0.2 (MINI) (33) and the Montgomery-Åsberg Depression Rating Scale (MADRS) (34). Interviews were conducted by psychological researchers with a master’s degree and at least 1 year of experience in clinical interviewing.

Based on the results of the first interview, some participants completed a second video interview session. This 30-minute session involved a board-certified psychiatrist specializing in geriatric psychiatry, who diagnosed patient status according to the Diagnostic and Statistical Manual of Mental Disorders, Fifth Edition (DSM-5) criteria. Participants diagnosed with major depressive disorder, persistent depressive disorder (dysthymia), or depressive disorder not otherwise specified (NOS), requiring referral to a local healthcare center, were classified as depressed. Participants who completed at least one video interview but were not diagnosed with depression requiring referral were classified as at risk of depression. Participants continued using *Hyodol* throughout the survey and interview periods, and their responses and usage logs from these periods were also included in the model development. Participants who did not complete the survey or interview process were excluded from analysis in accordance with a complete case analysis strategy.

### 2.4 Dataset and classification objective

The objective of the modeling was to screen for and identify community-dwelling older adults who were clinically diagnosed with depression by a psychiatrist and may require referral to a local healthcare provider. By identifying measurable and significant signals associated with depression risk, the prediction model may help identify older adults at risk of depression at an early stage and support timely further assessment, follow-up, and targeted care. To maximize model sensitivity in detecting clinically diagnosed cases within a limited and imbalanced dataset, the model was initially trained using a sensitive labeling scheme, classifying participants as either non-depressed or symptomatic (i.e., at-risk or depressed). Subsequently, the model’s screening utility was evaluated based on its performance in identifying individuals with clinical depression.

For model development and evaluation, participants were divided into two temporally distinct samples: Sample 1, comprising individuals who completed clinical surveys and video consultations between April and December 2024, and Sample 2, comprising participants who participated between January and June 2025. Sample 1 was used as the training set to classify either non-depressed participants or symptomatic participants. Sample 2 served as the test set to evaluate model performance for two tasks: (i) distinguishing depressed participants from the combined group of non-depressed and at-risk participants, and (ii) identifying symptomatic participants (at-risk and depressed) versus non-depressed participants. The first task was designed to reflect the model’s practical screening utility, aligning with real-world clinical objectives of identifying individuals who warrant further psychiatric evaluation or referral.

For modeling, the following features were selected and processed as the original candidate variables: ratios of positive, neutral, and negative sentiment responses to daily check-in questions; average daily frequencies of free conversation engagements and average word count of free conversation responses; average daily frequencies of patting, tapping, and holding hands with the robot; the average daily frequencies of medication and meal intake; and average daily frequencies of engagement with various content types (Bible or Buddhist scripture readings, storytelling, trot songs, English lessons, remembrance games, classical and religious music, exercise routines, quizzes, and hearing function checks). These features broadly reflect users’ active engagement and adherence levels, which are frequently used in digital sensing and digital phenotyping studies (35, 36) and have been reported to be helpful for predicting depression risk (36, 37) and digital intervention outcomes (38, 39).

Several demographic characteristics, including religion, educational level, marital status, and cohabitation status, were included as candidate features in binarized format: religion = 1 if the participant reported any religious affiliation; educational level = 1 if the participant had not completed education beyond high school; marital status = 1 if the participant was never married, divorced, or bereaved; and cohabitation status = 1 if the participant lived alone.

### 2.5 Model training and optimization

From the pool of candidate features, the final feature set was selected based on mutual information (MI) values, which were estimated across 1,000 bootstrapped samples to assess the strength of association between each feature and the training target variable (non-depressed vs. symptomatic individuals). Bootstrapping was used to reduce reliance on a single sample and to assess whether a feature remained informative across slightly different resampled datasets. For each feature, the percentage of iterations in which its MI value exceeded a threshold of 0.01 was calculated. This percentage, referred to as the feature inclusion rate, reflected how consistently each feature contributed predictive information. The inclusion rate was then used as a feature-stability criterion and treated as a hyperparameter during model tuning, along with other model-specific parameters. This procedure ensured that the final model relied on consistently informative features, while also identifying the optimal inclusion rate threshold from a predictive perspective.

A Random Forest classifier was employed as the core model for binary classification. Random Forest is one of the most widely used and well-established models to predict mental health symptoms including depression and is known to effectively capture complex, non-linear relationships and interactions among features without requiring strong parametric assumptions (40, 41). Research indicates that ensemble models such as Random Forest exhibit better performance for predicting depression in older adults compared to other machine learning models (42), although other work found no clear advantage in predicting depression over other models such as logistic regression (41). Model optimization was conducted using RandomizedSearchCV, which simultaneously tuned the MI-based feature inclusion threshold and other model hyperparameters. The random search procedure prioritized sensitivity (recall) to enhance the model’s screening capability and was performed over 100 iterations with five-fold stratified cross-validation based on the training target variable. The model-specific hyperparameters explored during the optimization process included the number of estimators, maximum tree depth, maximum number of features considered at each split, minimum samples required to split an internal node, minimum samples required at a leaf node, class weights, and the complexity parameter for minimal cost-complexity pruning.

After fixing the final feature set and hyperparameters, the classification probability threshold was optimized by sweeping cut-offs from 0.05 to 0.50 in 0.05-point increments. For each candidate cut-off, performance metrics, including sensitivity and specificity, were evaluated using five-fold stratified cross-validation on the training data. We first calculated Youden’s J statistic (sensitivity + specificity − 1) for each threshold and used the cut-off with the highest J value as the primary candidate, as this criterion is commonly used in diagnostic and screening research to identify a balanced operating point on the ROC curve (43). However, because Youden’s J weights sensitivity and specificity equally, it was not used in isolation. Instead, the candidate threshold was subsequently reviewed together with sensitivity and specificity in the training set to ensure that the final cut-off reflected a balanced and clinically interpretable trade-off for screening use. Based on this procedure, a threshold of 0.40 was selected for the final Random Forest model, together with the finalized feature set and optimized hyperparameters.

### 2.6 Evaluation and interpretation

Final testing was conducted by applying the fixed final model, trained on Sample 1 using sensitivity-maximizing binary labels (non-depressed vs. symptomatic), to the independent Sample 2 to evaluate its external validity and generalizability to new participants. To assess the model’s screening capability for individuals with clinical depression, sensitivity, accuracy, specificity, precision, and F1 score were computed based on clinically determined depression status, as established by a board-certified psychiatrist. This evaluation examined the model’s clinical utility in distinguishing individuals with clinical depression from all others (non-depressed and at-risk). Additionally, the model’s performance in detecting symptomatic individuals (at-risk and depressed) was assessed. All model training and evaluation were conducted using a fixed random seed of 42 to ensure reproducibility. Finally, Shapley Additive Explanation (SHAP) values were calculated for the selected features to identify their relative importance in both the training and test procedures. Analyses were implemented in Python using the scikit-learn library (44).

## 3 Results

### 3.1 Participants

Of the 443 participants who enrolled in the clinical survey and video consultation sessions, 215 participants who completed the process and were classified as non-depressed, at-risk, or depressed were included in the study, comprising 170 in Sample 1 and 45 in Sample 2. The mean age of all participants was 80.6 ± 5.44 years (Sample 1: 80.4 ± 5.57; Sample 2: 81.5 ± 4.84), and 87% were female (Sample 1: 88%; Sample 2: 84%). Overall, 95% of participants had completed high school or higher education (Sample 1: 95%; Sample 2: 93%). In Sample 1, 122 participants (72%) were classified as non-depressed, 34 (20%) as at-risk, and 14 (8%) as depressed. In Sample 2, 12 participants (27%) were classified as non-depressed, 23 (51%) as at-risk, and 10 (22%) as depressed.

### 3.2 Feature selection and hyperparameter optimization

Through MI-based feature selection and iterated random search, the following hyperparameters were selected for the final model: 200 trees, a maximum depth of 20, no restriction on the number of features, a minimum of 10 samples to split an internal node, 8 samples per leaf, balanced subsample class weights, and a minimal-cost-complexity pruning parameter of 0.001. An MI-based feature inclusion rate of 0.45 was selected. The features included in the model were: educational level, the ratio of positive sentiment responses to daily check-in questions, the daily frequency of free conversations, the frequency of patting the robot, the frequency of tapping the robot, the frequency of meal intake, and the frequency of engaging with the quiz content.

### 3.3 Classification threshold determination

**Figure 1:**
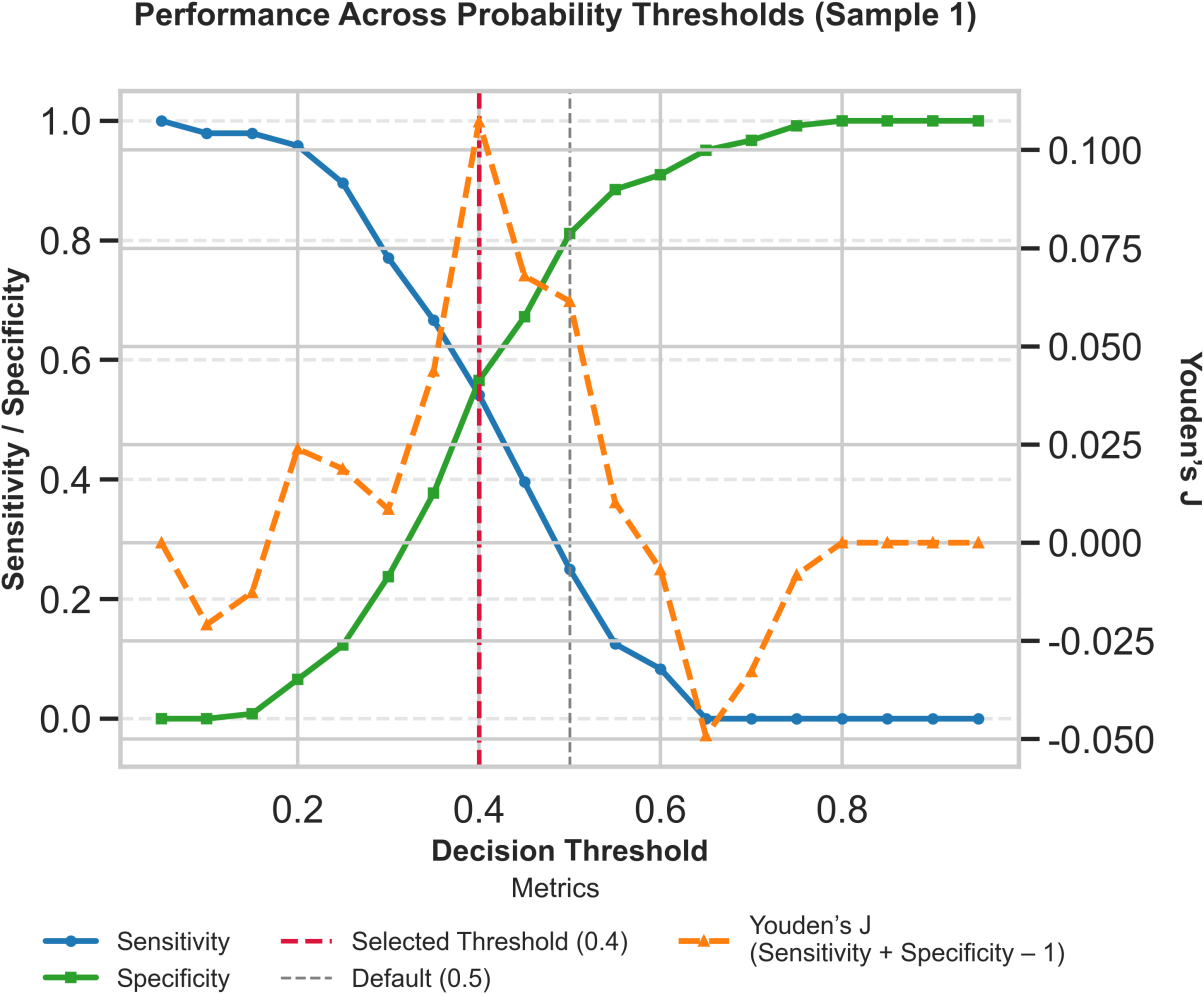
Comparison of model sensitivity, specificity, and Youden’s J statistic across probability thresholds.

The performance of the optimized Random Forest model across probability thresholds ranging from 0.05 to 0.50 is summarized in **Figure 1**. Youden’s J statistic was highest (0.11) at a threshold of 0.40, indicating the optimal trade-off between identifying at-risk individuals and minimizing false negatives. This threshold was therefore adopted for subsequent validation.

### 3.4 Evaluation of screening utility

Prediction scores for symptomatic participants (i.e., at-risk and depressed) and for depressed participants (vs. non-depressed and at-risk) in Sample 2 are summarized in **Table 1**. The current model identified symptomatic participants with a sensitivity of 0.939 and depressed participants with a sensitivity of 0.900. Specifically, the model missed only 2 out of 33 symptomatic cases and 1 out of 10 depressed cases. However, it also misclassified 9 non-symptomatic participants as symptomatic and 28 non-depressed participants as depressed, resulting in false positive ratios of 0.750 and 0.800, respectively. This outcome suggests that the probability threshold optimized during training may have been too lenient for this sample, leading to an elevated false-positive rate. These findings indicate that, while the model effectively prioritizes sensitivity as intended for a screening-oriented framework, its specificity may require recalibration when applied to new populations to minimize unnecessary referrals in real-world settings.

**Table 1:** Classification performance of the depression-risk model.

Category	Symptomatic (vs. non-depressed)	Depressed (vs. at risk + non-depressed)
Sample composition		
Actual positive, *n*	33	10
Actual negative, *n*	12	35
Performance metrics		
Sensitivity	0.939	0.900
Specificity	0.250	0.200
Accuracy	0.756	0.356
Precision	0.775	0.243
F1 score	0.849	0.383

Performance testings are based on testing in Sample 2 (*N* = 45) using the model trained on Sample 1. Symptomatic participants include individuals at risk of depression and those diagnosed with significant depression requiring referral to local healthcare centers. Participants with depression refer exclusively to those with a clinically significant diagnosis of depression.

### 3.5 Feature interpretation

**Figure 2:**
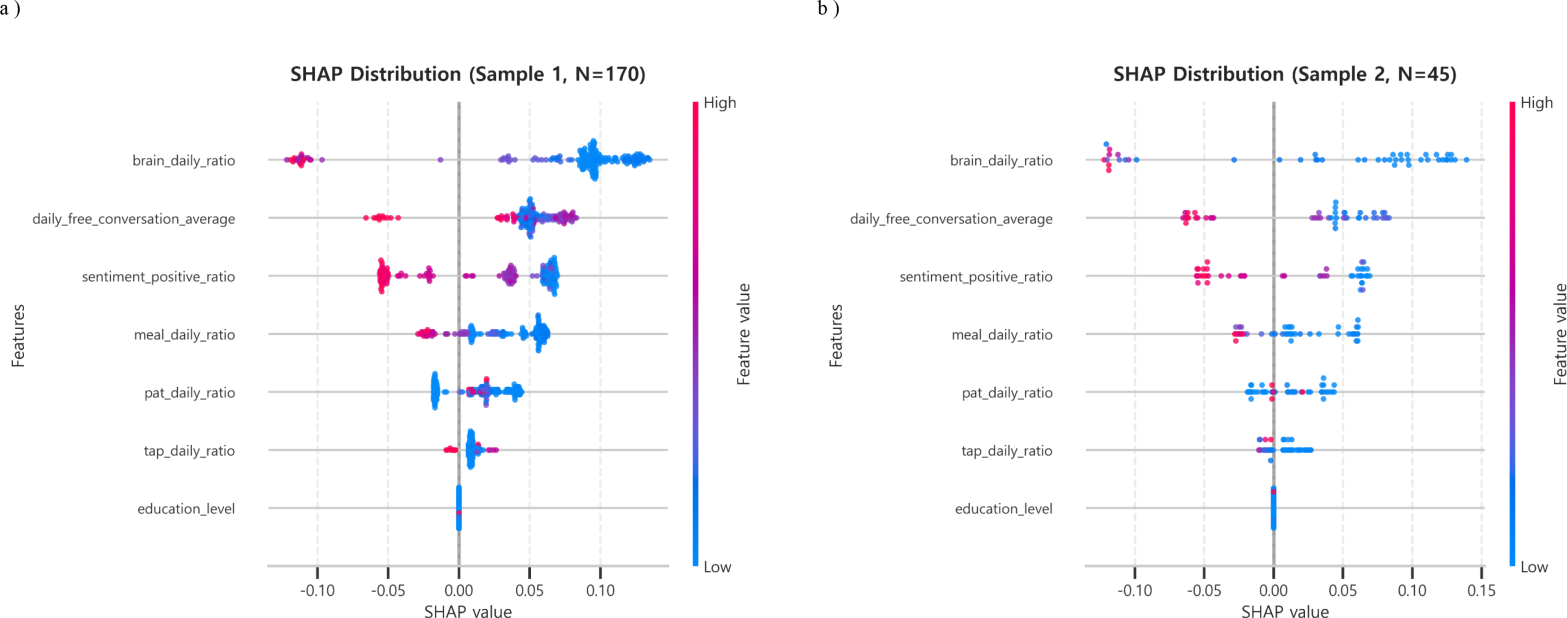
SHAP summary plots for **(a)** training set and **(b)** test set. *Feature labels*: brain_daily_ratio (the frequency of engaging in the quiz content), daily_free_conversation_average (the daily frequency of free conversations), sentiment_positive_ratio (the ratio of positive sentiment responses to daily health check-in questions), meal_daily_ratio (the frequency of meal intake), pat_daily_ratio (the frequency of patting the robot), tap_daily_ratio (the frequency of tapping the robot).

**Figure 2** illustrates the distributions of SHAP values for each included feature across the training (**Figure 2a**) and test sets (**Figure 2b**). SHAP analyses demonstrated high consistency between the two datasets, indicating that the model relied on similar feature patterns when making predictions, thereby supporting its generalizability. Overall, participant engagement with quiz content emerged as the strongest predictor of depression, followed by engagement in free conversations, underscoring the importance of interactive communication with the robot in identifying depressive symptoms. In addition, the ratios of positive responses to daily check-in questions and regular meal intake were influential predictors, along with physical interaction frequency (e.g., patting or tapping the robot), highlighting the relevance of both emotional engagement and behavioral regularity in distinguishing participants at risk for depression.

## 4 Discussion

The present study was an exploratory investigation aimed at detecting depression risk among older adults using the SAR *Hyodol*, enabling the identification of individuals at risk of depression solely through robot interaction without the involvement of human caregivers. The development of this model may facilitate the early identification of at-risk individuals and those requiring timely care at local healthcare centers, thereby supplementing primary medical services and potentially reducing the costs associated with the delayed detection of depression (45, 46).

Overall, the preliminary analysis yielded encouraging results. The model demonstrated high sensitivity in detecting both individuals at risk of depression and those requiring referral to local healthcare centers, indicating its potential utility as a screening tool for depression risk. This screening tool can be particularly valuable because it automatically assesses users’ depression using passive usage data, eliminating the need for active input from users or caregivers. Consequently, it may reduce the burden on older adults of continuously responding to survey questionnaires and alleviate the workload of therapists conducting active assessments. However, the high rate of false positives and low specificity suggest that the tool may generate additional costs due to false alarms and unnecessary follow-up procedures.

At its current stage, the tool appears best suited as an adjunct for the rapid identification of older adults who may be at risk for depression, rather than as a standalone diagnostic solution, and any positive screening result should be followed by additional clinical assessment or follow-up screening before intervention is considered to minimize unnecessary costs associated with false positives. In practice, the model could be implemented within a stepwise workflow. First, the model would operate in the background using routinely collected interaction data and generate a risk flag only when the predicted probability exceeds a prespecified screening threshold. Second, instead of triggering direct referral or treatment, the flag would prompt a low-burden secondary review, such as a brief phone check-in by a welfare-center staff member, confirmation of recent functional or emotional change by a caregiver, or administration of a short repeat symptom screener. Third, only individuals who remain concerning after this secondary step would proceed to more formal clinical evaluation or referral. Under such a workflow, false positives would primarily lead to additional monitoring or brief follow-up rather than unnecessary treatment, thereby reducing the practical harm of low specificity. This approach may be feasible when sufficient medical and community healthcare resources are available, but in resource-limited settings, *Hyodol* may need to identify high-risk individuals more selectively to reduce screening costs. This will require further improvement of the model to reduce false positives and enhance its practical utility in resource-constrained care settings.

SHAP analysis was conducted on both the training and test sets to further interpret the model’s behavior, revealing consistent feature contributions across datasets. The most influential predictors of depressive status were participant engagement with quiz content and conversational interactions with the robot. These findings align with previous research, indicating that cognitively stimulating and socially interactive activities supported by robots can mitigate depressive symptoms in older adults (47, 48). Additional key predictors included the ratio of positive responses to daily check-in questions and the regularity of meal intake, reflecting domains of emotional well-being and behavioral consistency that are often disrupted in late-life depression. This finding is consistent with prior evidence linking irregular routines, such as inconsistent meal schedules, to increased depression risk in older adults (48, 49). Physical interactions with the robot (e.g., patting or tapping) were also predictive of depressive status, echoing findings from therapeutic robot studies (e.g., *PARO*) in which affectionate touch is associated with mood improvements and reduced loneliness (50). Collectively, these findings suggest that socially assistive robots can serve not only as companions or functional aids, but also as tools capable of capturing meaningful behavioral markers of mental health risk, thereby reinforcing their utility for community-based screening.

However, as this study is predictive rather than causal in nature, the identified factors should be interpreted as potential markers of depression, and strong causal inferences should be made with caution. Several limitations of the study should also be acknowledged. One notable limitation of this study is the lack of incorporation of the actual content of participants’ verbal responses to daily check-ins or free conversations. The current analysis relied solely on dialogue frequency and sentiment, without incorporating richer semantic and paralinguistic information from voice data, such as speech content and acoustic features including pitch, articulation, pause duration, jitter, and shimmer (51). Moreover, enhancing the system’s capacity to detect acute psychological risks—including suicidality—and to deliver timely alerts would substantially strengthen the clinical utility of *Hyodol*. Incorporating these capabilities would advance the integration of medical AI and IoT technologies into SARs for mental health monitoring.

Finally, the model’s generalizability may be limited for several reasons. In particular, the sample was small in size and not fully representative of the broader older-adult population, as the study included participants who were already using *Hyodol* rather than a randomly selected sample of community-dwelling older adults. A higher false positive rate observed in the test set can also limit generalizability. This limitation is partially attributable to the unbalanced distribution of depression status between the two samples. Sample 2 included relatively more symptomatic participants than Sample 1, likely reflecting differences in participant characteristics between those recruited in 2024 and 2025. Consequently, the probability threshold determined based on Sample 1 was too low when applied to Sample 2, resulting in inflated sensitivity but reduced specificity.

Therefore, future studies may benefit from technical strategies such as threshold recalibration, probability calibration, and flexible yet generalizable domain adaptation approaches to better accommodate inter-sample differences and distribution shifts (52). Additionally, incorporating richer multimodal predictors, such as conversational patterns and acoustic features, may improve model specificity beyond the current interaction-frequency-based variables alone. Also, further validation is necessary among older adults in diverse regions and usage settings to assess whether this detection tool is truly generalizable and whether its sensitivity can be consistently reproduced. We aim to extend this work to more diverse samples to further evaluate *Hyodol*’s clinical effects, develop more advanced models with improved specificity by incorporating response content and acoustic features, and investigate whether daily symptom changes or response patterns at specific time points may indicate elevated risk of depression or suicidality, with the long-term goal of enabling more timely responses to mental health risk.

## Data Availability

The datasets presented in this article are not readily available because they contain privacy-sensitive information. Requests to access the datasets should be directed to the corresponding author.
